# The associations between social, built and geophysical environment and age-specific dementia mortality among older adults in a high-density Asian city

**DOI:** 10.1186/s12942-020-00252-y

**Published:** 2020-12-04

**Authors:** Hung Chak Ho, Kenneth N. K. Fong, Ta-Chien Chan, Yuan Shi

**Affiliations:** 1 Department of Urban Planning and Design, The University of Hong Kong, Hong Kong, China; 2 Department of Rehabilitation Sciences, The Hong Kong Polytechnic University, Hong Kong, China; 3 Research Center for Humanities and Social Sciences, Academia Sinica, Taipei, Taiwan; 4 Institute of Future Cities, The Chinese University of Hong Kong, Hong Kong, China

**Keywords:** Dementia, Mortality, Built environment, Social environment, Geophysical environment, Age-specific

## Abstract

**Background:**

Although socio-environmental factors which may affect dementia have widely been studied, the mortality of dementia and socio-environmental relationships among older adults have seldom been discussed.

**Method:**

A retrospective, observational study based on territory-wide register-based data was conducted to evaluate the relationships of four individual-level social measures, two community-level social measures, six short-term (temporally varying) environmental measures, and four long-term (spatially varying) environmental measures with dementia mortality among older adults in a high-density Asian city (Hong Kong), for the following decedents: (1) all deaths: age >= 65, (2) “old-old”: age > = 85, (3) “mid-old”: aged 75–84, and (4) “young-old”: aged 65–74.

**Results:**

This study identified 5438 deaths (3771 old-old; 1439 mid-old; 228 young-old) from dementia out of 228,600 all-cause deaths among older adults in Hong Kong between 2007 and 2014. Generally, regional air pollution, being unmarried or female, older age, and *daily O*_*3*_ were associated with higher dementia mortality, while more urban compactness and greenness were linked to lower dementia mortality among older adults. Specifically, being unmarried and the age effect were associated with higher dementia mortality among the “old-old”, “mid-old” and “young-old”. Regional air pollution was linked to increased dementia mortality, while urban compactness and greenness were associated with lower dementia mortality among the “old-old” and “mid-old”. Higher *daily O*_*3*_ had higher dementia mortality, while districts with a greater percentage of residents whose native language is not Cantonese were linked to lower dementia mortality among the “old-old”. Economic inactivity was associated with increased dementia mortality among the “young-old”. Gender effect varied by age.

**Conclusion:**

The difference in strengths of association of various factors with dementia mortality among different age groups implies the need for a comprehensive framework for community health planning. In particular, strategies for air quality control, usage of greenspace and social space, and activity engagement to reduce vulnerability at all ages are warranted.

## Introduction

Dementia is a common disorder among the ageing population, particularly in developed countries [2, 18]. Previous studies have indicated an association between demographic structure and dementia [21, 61, 64]. For example, older people are a major vulnerable population with high prevalence of dementia, especially those with an unhealthy diet, low mobility and a low level of physical activity [5, 47]. As activities of daily living as well as mobility could affect both dementia and quality of life [5], numerous studies have also investigated the linkage between dementia and quality of life [3, 7, 35], specifically in the local context of how physical activities improve the wellbeing of older people with this disease [1, 57]. In addition, human behaviors, activities of daily living and spatial mobility of older adults could also be affected by the environment surrounding their residences [10]; therefore, this has also led to a research trend of investigating influences of built and social environments on the prevalence of dementia [70]. For example, recent studies have found that green space as a built environmental factor reduces dementia and its associated diseases [19, 67, 70]. Some studies have also documented the adverse impacts of socioeconomic and racial problems on these diseases [8, 15, 17, 63, 71], and other studies have found that long-term air pollution (e.g. traffic-related air pollution) can negatively influence cognitive function [12, 13, 25, 41, 49, 52, 60, 73]. There are also studies describing the short-term risks of dementia, due to extreme weather or daily variation of temperature, and air quality [32, 38, 66]. In the above-summarized literature, detailed linkages between exposure to such factors and dementia have been discovered.

Despite the research trends above, the fatal effects of dementia and its relationships with the social and geophysical environment have rarely been discussed, especially in the context of a high-density Asian city. Although mortality associated with dementia may not be as high as other chronic diseases such as respiratory diseases and cancers, there are still remarkable death counts. According to the 2016 Global Burden of Disease Study [48], dementia was the fifth leading cause of death globally, accounting for 2.4 million deaths. Mortality due to dementia may also be significant in high-density Asian cities. For example, approximately 2.1% of male deaths and 4.0% of female deaths were from dementia in 2016, based on Public Health Information and Statistics of Hong Kong (https://www.healthyhk.gov.hk). Furthermore, dementia was one of the two brain-related diseases recognized among the “ten leading causes of death” for both males and females in the above database. This indicates that dementia can be a significant factor causing fatal effects to those who are older ages and whose dementia is not due to genetic issues. While it is still important to evaluate the “survival population” (the population living with dementia), it is also essential to study dementia mortality in order to better develop protocols for health surveillance and community planning.

Furthermore, as Hong Kong is a city with high-density living and a compact environment [34, 51], it is expected that “population stress” has been occurring due to the local population growing faster than the available land [14]. Based on the perspective of human ecology, this population growth can also influence the change of urban form and natural environment [16]. Therefore, it is important to enhance the understanding of the human–environment nexus across a high-density city, in order to create a “healthy city” that can protect all ages and all vulnerable populations [29, 69], including people with dementia at different ages.

We hereby develop a retrospective, observational, cross-sectional design based on territory-wide register-based data to evaluate the potential influences of social and environmental burdens on dementia mortality among older adults in a high-density Asian city (Hong Kong). The objectives of this study include evaluating the links between age-specific dementia mortality among older adults in Hong Kong and (1) individual-level socioeconomic characteristics, (2) community-level socioeconomic characteristics, (3) short-term environmental changes, and (4) long-term environmental deprivation.

## Methods

### Conceptual framework

**Fig. 1:**
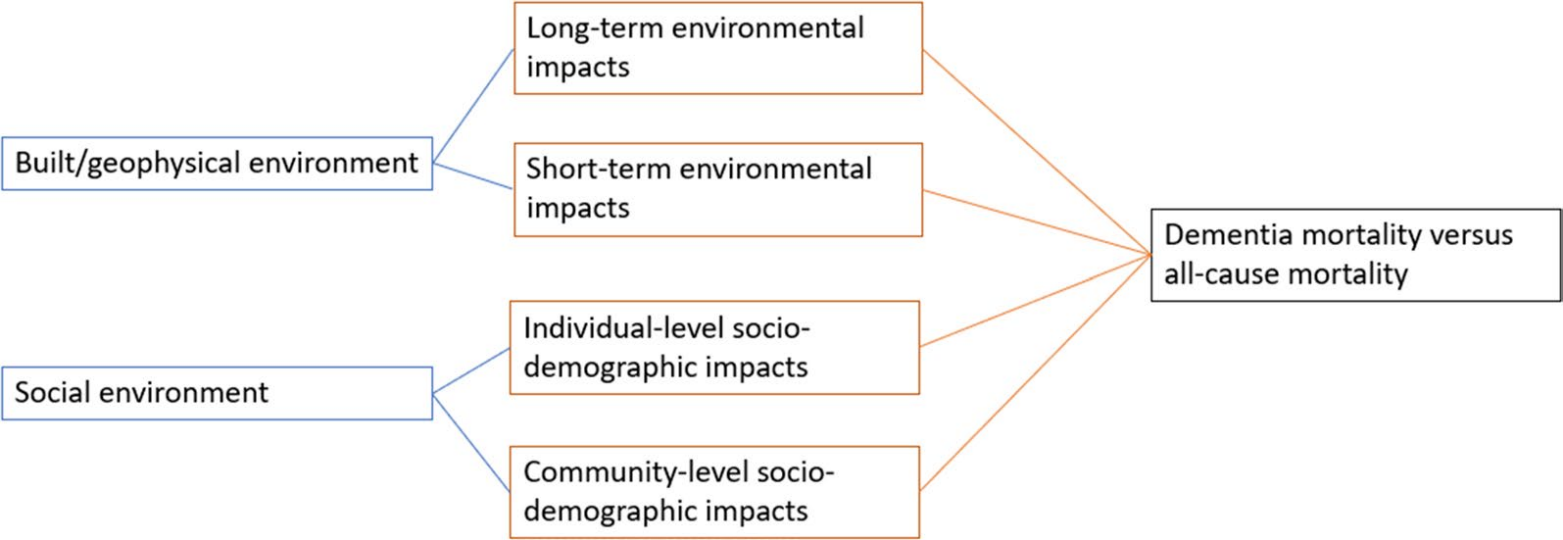
Conceptual framework

This study hypothesized four types of environmental influences on mortality (Fig. 1): individual-level socio-demographic impacts, community-level socio-demographic impacts, long-term environmental impacts, and short-term environmental impacts. In general, individual-level socio-demographic impacts and community-level socio-demographic impacts are related to influences from the social environment. Long- and short-term environmental impacts are related to effects from the built and geophysical environment. All of the risk factors from each dimension could influence mortality independently. Specifically, some of the risk factors may create higher impacts on dementia mortality than deaths from other causes in various age groups. Based on this framework, the following measures were used in statistical modelling.

### Health outcome variable

This study first selected persons who died from dementia as “cases” and all-cause deaths as “controls” for comparison. The reason for using all-cause deaths as controls was to evaluate whether dementia could be more fatal in situations with various socio-environmental impacts, in comparison with the “normal scenario” (based on all-cause deaths).

The Hong Kong Census and Statistics Department’s mortality dataset (2007–2014) was applied [33, 43], with details of all decedents recording the following information: (1) date of death; (2) age; (3) gender; (4) occupation; (5) marital status; (6) cause of death registered based on the 10th version of the International Statistical Classification of Diseases and Related Health Problems (ICD-10); and (7) location of residence registered at the level of “tertiary planning unit” (TPU), which is a fine spatial unit for regional planning with vital statistics in Hong Kong. Specifically, there were 287 TPUs within the total land of Hong Kong (area: 1106 km^2^) in 2006. Decedents with dementia were retrieved based on the following ICD-codes: ICD-10 F00–F03.

### Individual-level socio-demographic measures

A binary measure of gender with 1 for male and 0 for female was classified based on the personal information of each decedent. A binary measure of economically inactive was also classified based on the information of occupation, with 1 for decedents who were “economically inactive” and 0 representing other decedents. This measure represented the socioeconomic status of each decedent, based on whether the decedent was unemployed or did not participate in any type of job. A binary measure for marital status was used, with 1 for “unmarried” and 0 for “married” to indicate potential social isolation of each decedent. Furthermore, age was recorded as a continuous variable. These individual-level social factors are related to prevalence of dementia [8].

### Community-level socio-demographic measures

**Fig. 2:**
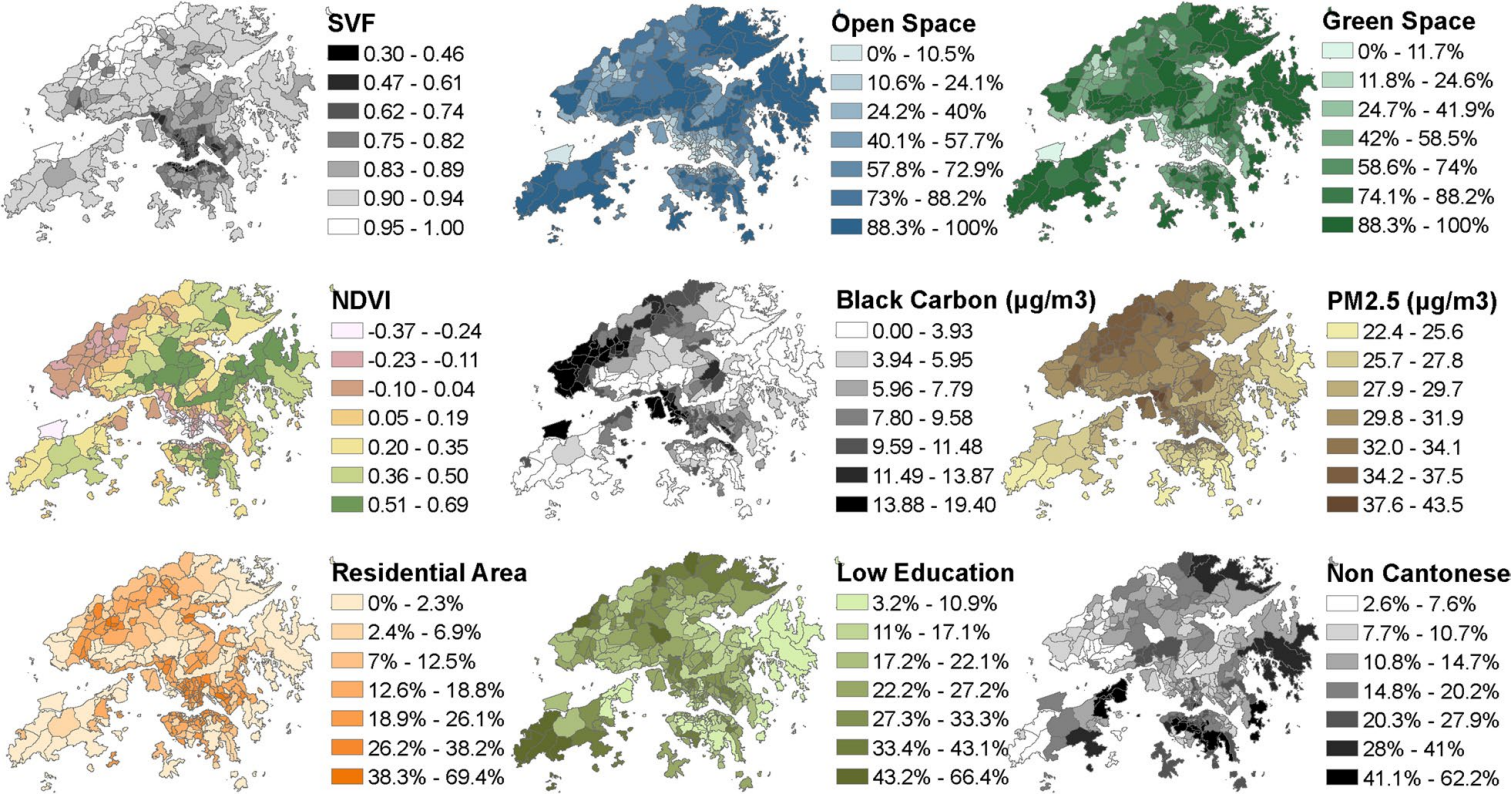
Spatial distribution of community-level socio-environmental factors

Community-level socio-demographic measures (Fig. 2) were spatially varying variables obtained from the 2006 Hong Kong census data recorded at the TPU level, including (1) percent of low-education population (*Low education %*), and (2) percent of the population whose native language is not Cantonese (*Not Cantonese %*). In this study, *Low education %* was defined as the percentage of people with a primary school education or less. This measure represents a community with low preparedness for health risk due to the level of education. Since the native dialect of the local population is Cantonese, a high percentage of *Not Cantonese %* indicates a neighbourhood with potentially mixed cultures (e.g. cultural heritage of migrants, and a district with visible minorities), which may suffer more problems associated with community health. These community-level socio-demographic measures have been documented to be associated with dementia [45, 56].

### Measures of long-term environmental impacts

Two spatially varying measures related to long-term impacts of the built environment (Fig. 2), which may influence the prevalence of dementia [19, 67, 70], were used in this study: (1) *urban compactness*, and (2) *greenness*.

Specifically, *urban compactness* is calculated from an urban sky view factor (SVF) dataset commonly used in local studies [58]. SVF is an indicator of unobstructed sky across the urban area ranging from 0 to 1, in which a lower value can represent an area with more obstructed sky due to a high-density built environment, and a higher value can indicate an area with less obstructed sky. This study first obtained the average SVF of each TPU and matched the average values to all decedents based on their locations of residence. A measure of building density representing a “decrease in percentage of sky view” (*percent of no sky view*) was calculated by the following equation: (1 − average SVF) × 100%.

*Greenness* was estimated by the average normalized difference vegetation index (NDVI) of each TPU. NDVI is a spectral index derived by the infrared and red bands of a satellite image [62, 72], with a range between − 1 and 1. In theory, a low value indicates an area with less greenness and a high value represents an area with more greenness. In this study, the NDVI map was a resampled spatial dataset with 15 m resolution estimated by an IKONOS multispectral image. Since NDVI itself was unitless, we defined a 0.1 increase of NDVI as the unit for the measure of greenness.

Air pollution is mainly contributed by the following two sources: regional and local (traffic-related). Therefore, this study applied two spatially varying measures of air pollution related to long-term impacts of the geophysical environment (Fig. 2): (1) *regional air pollution*, and (2) *traffic*-*related air pollution*. Several studies have found associations between these measures and dementia [12, 13, 25, 33, 41, 49, 52, 60, 73]. Additionally, a local study has found that approximately 50% of air pollution was regional while 50% was related to traffic emissions [6]. In this study, *regional air pollution* was represented by the spatial distribution of fine particulate matters. *Traffic*-*related air pollution* was represented by the spatial distribution of black carbon. Since air quality maps from land use regression [59] have commonly been used to estimate long-term impacts on health risk caused by spatial variation of air quality, we applied hybrid results of land use regression and field measurements operated by the Hong Kong Environmental Protection Department (EPD) from previous studies [4, 40] to represent spatial distributions of regional air pollution and traffic-related air pollution across Hong Kong. These datasets were originally in 10 m resolution and were spatially averaged based on the TPU boundary to obtain the measures for this register-based study. An average increase of 10 µg/m^3^ in each air pollutant across a sub-district was used as the unit of the measures above.

### Measures of short-term environmental impacts

Previous studies have indicated the potential influences of short-term environmental changes on dementia [32, 38, 66]. Therefore, this study applied six temporally varying measures (without spatial information) related to short-term environmental impacts, including: (1) influence of hot weather (*Hot day*), (2) influence of cold weather (*Cold day*), (3) daily variation of respirable suspended particulates (*daily PM*_*10*_), 4) daily variation of nitrogen oxides (*daily NO*_*x*_), 5) daily variation of tropospheric ozone (*daily O*_*3*_), and 6) daily variation of humidity (*daily humidity*). Based on previous local studies in Hong Kong [11, 23, 68], the above factors have immediate effect. Some studies also showed that air pollution has the strongest impact during lag 0 day [54]. Therefore, a lag 0 day scenario was used in this research.

*Hot day* is a binary measure calculated based on the daily average temperature at the headquarters of HKO, with “1” indicating a day with temperature > = 95th percentile (based on long-term weather records between Jan. 1, 1971 and Aug. 31, 2015) and “0” indicating other days. *Cold day* is a binary measure indicating days with temperature <= 5th percentile as “1” and the other days as “0”. *Daily humidity* is calculated based on the daily average of humidity obtained from the weather station located at the headquarters of HKO.

*Daily PM*_*10*_, *daily NO*_*x*_, and *daily O*_*3*_ are the daily average of seven non-roadside stations operated by EPD (Central Western, Sham Shui Po, Sha Tin, Tai Po, Tsuen Wan, Kwai Chung, and Tap Mun). These seven stations are located in either urban districts or the countryside in Hong Kong. Using the daily average of these seven non-roadside stations to estimate short-term impacts of air pollution in Hong Kong for the purpose of excluding the bias of intra-city spatial variations in estimation has been documented elsewhere [29–32]. A 10 µg/m^3^ increase in each air pollutant for a day was used as the unit of the measures for air quality.

### Statistical analysis

Binomial logistic regression was applied to estimate the potential impacts of social, built and geophysical environment on dementia decedents aged > = 65, with the use of the *glm* package in *R* software. Odds ratios (OR) and 95% confidence intervals (CI) estimated by independent effects of all measures are reported.

Subgroup analysis was then applied to estimate the associations of the socio-environmental factors with mortality at different ages. Decedents were stratified as follows: (1) “old–old”, aged > = 85, (2) “mid-old”, aged between 75 and 84, and (3) “young-old”, aged between 65 and 74. We re-analyzed the links between mortality and the social, built and geophysical environment for these four subgroups of decedents based on binomial logistic regression with the same model structure.

In order to avoid multicollinearity, preliminary variance inflation factor (VIF) analyses have been applied to the above regressions. Based on preliminary tests, VIFs for all independent variables in each regression were lower than 3, indicating the suitability of applying all variables as co-variates in this study.

## Results

### Data summary

Listwise deletion for missing death date or missing location of residence was first performed for retrieving the analytic dataset, as these data are missing completely at random. After exclusion of decedents with a missing death date or missing location of residence, the analytic dataset included 5438 deaths of older adults who died from dementia. There were 3771 old-old deaths from dementia. Among the mid-old decedents, there were 1439 deaths from dementia. There were also 228 young-old decedents who succumbed to dementia. In general, the ratio between dementia and all-cause deaths increased as age increased (Table 1).

**Table 1:** Number of deaths associated with dementia mortality and all-cause mortality among each sub-population

Decedents	Dementia	All-cause
Old-old	3771	91,064
Mid-old	1439	92,158
Young-old	228	45,378
All ages	5438	228,600

Based on a summary of socio-environmental characteristics (Table 2), deaths in Hong Kong tended to occur among those living in a district with long-term impact of regional air pollution much higher than the standard of the World Health Organization (WHO). According to the WHO air quality guidelines, the acceptable annual mean of fine particulate matters (PM_2.5_) should be lower than 10 μg/m^3^. However, deaths in Hong Kong were associated with PM_2.5_ levels at more than three times the threshold, while dementia deaths were exposed to a slightly higher level than all-cause deaths.

**Table 2:** Data summary of the analytical dataset

Dementia deaths	Dementia deaths				All-cause deaths			
	All decedents	Old-old	Mid-old	Young-old	All decedents	Old-old	Mid-old	Young-old
Regional air pollution (µg/m^3^)	32.9	32.9	33.1	32.7	32.4	32.4	32.5	32.5
Traffic-related air pollution (µg/m^3^)	9.6	9.6	9.7	9.4	9.2	9.2	9.2	9.3
Day with high temperature (%)	7.2	7.3	6.9	8.8	7.0	7.0	7.1	7.1
Day with low temperature (%)	5.9	6.0	6.3	3.1	6.4	6.8	6.3	6.2
Daily PM_10_ (µg/m^3^)	46.3	46.3	46.7	43.6	47.9	47.8	48.0	47.8
Daily NO_x_ (µg/m^3^)	90.4	90.7	89.7	91.2	93.1	93.1	93.1	92.9
Daily O_3_ (µg/m^3^)	42.4	42.6	42.2	40.7	41.7	41.8	41.8	41.4
RH	78.3	78.3	78.4	78.7	77.9	77.9	77.8	77.8
Percent of no sky view (%)	28.4	28.3	28.9	27.5	29.0	29.3	28.9	28.4
NDVI	− 0.05	− 0.05	− 0.06	− 0.03	− 0.03	− 0.03	− 0.03	− 0.02
Percent of low education (%)	26.8	26.7	27.0	26.8	26.8	32.7	27.2	27.2
Percent of non-Cantonese (%)	11.6	11.7	11.5	11.7	11.8	12.3	11.6	11.4
Economically inactive (%)	93.9	94.1	93.5	93.9	93.9	94.8	94.3	91.2
Unmarried (%)	75.7	81.6	63.6	56.1	59.3	73.9	53.5	41.4
Age	88.0	91.8	80.5	71.1	82.1	90.3	79.8	70.2
Male (%)	39.2	30.7	55.2	76.8	53.3	39.9	59.0	68.7

Furthermore, daily PM_10_ and O_3_ experienced by the dementia decedents almost reached the threshold of the daily mean based on the WHO guidelines. In addition, most of the dementia deaths were among the unmarried and economically inactive, and among those living in a district with a fairly high percentage of low education population.

### Socio-environmental impacts on all decedents

**Fig. 3:**
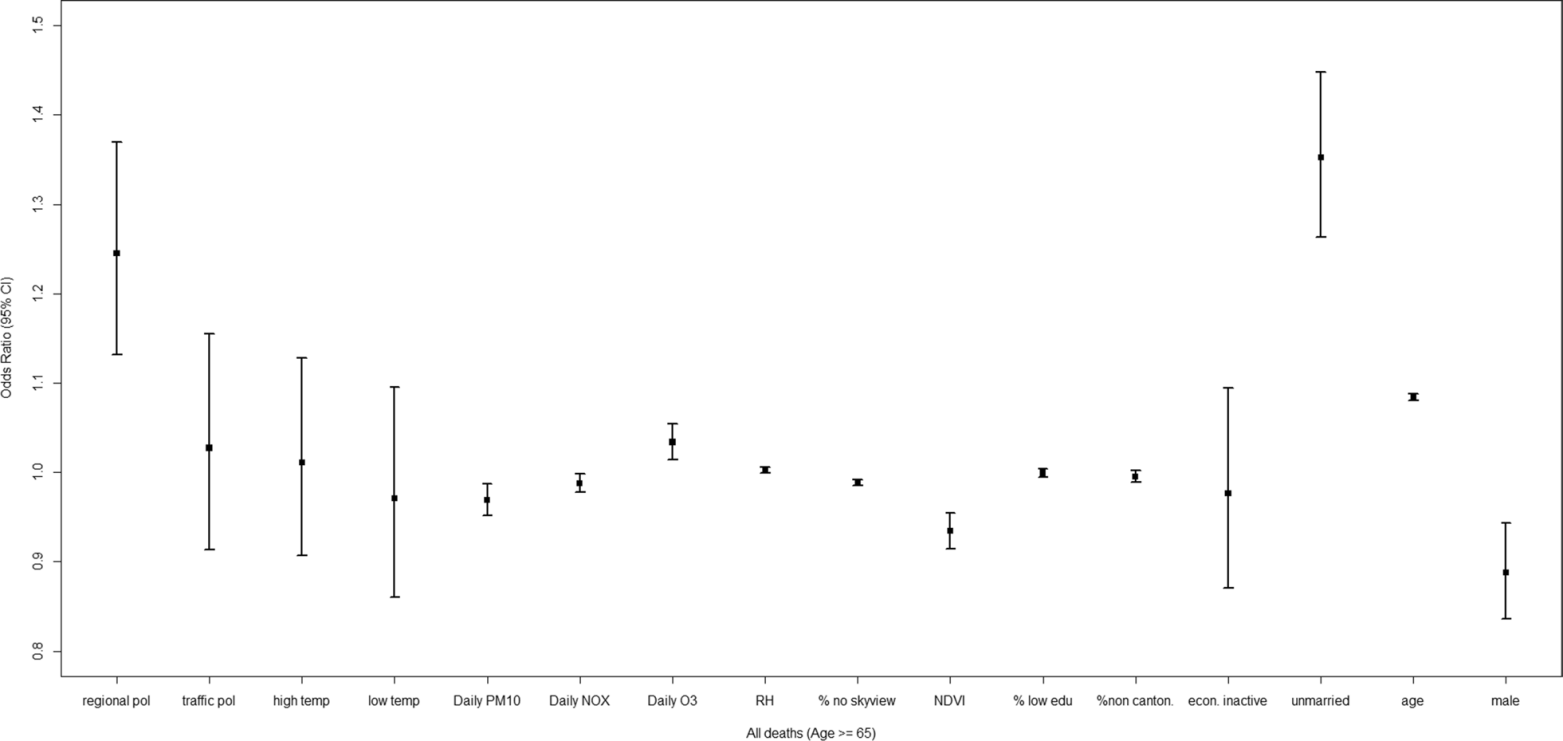
Adjusted odds ratio (OR) and the 95% confidence interval (CI) for the evaluation of relative impacts of social, built and geophysical environment on dementia mortality among all decedents (age > = 65)

Among all decedents who were older than 65 (Fig. 3), long-term exposure to regional air pollution was linked to higher dementia mortality (OR 1.245 [1.132, 1.369]) when compared with all-cause mortality, controlling for other social and environmental factors (Table 3). Being female and older age were also factors associated with higher dementia mortality.

**Table 3:** Adjusted odds ratio (OR) and the 95% confidence interval (CI) for the evaluation of relative impacts of social, built and geophysical environment on age-specific dementia mortality

	Odds ratio			
	All decedents	Old-old	Mid-old	Young-old
Regional air pollution (in 10 µg/m^3^)	*1.245 [1.132, 1.369]*	*1.186 [1.057, 1.330]*	*1.370 [1.142, 1.643]*	1.294 [0.817, 2.049]
Traffic-related air pollution (in 10 µg/m^3^)	1.027 [0.914, 1.155]	1.080 [0.937, 1.244]	0.966 [0.770, 1.212]	0.778 [0.448, 1.350]
Day with high temperature	1.011 [0.907, 1.128]	1.018 [0.892, 1.161]	0.969 [0.783, 1.199]	1.096 [0.675, 1.780]
Day with low temperature	0.971 [0.860, 1.095]	0.957 [0.828, 1.106]	1.073 [0.866, 1.347]	0.548 [0.252, 1.192]
Daily PM_10_ (in 10 µg/m^3^)	*0.969 [0.951, 0.987]*	*0.963 [0.941, 0.985]*	0.993 [0.962, 1.025]	*0.880 [0.792, 0.977]*
Daily NO_x_ (in 10 µg/m^3^)	*0.988 [0.977, 0.998]*	0.993 [0.980, 1.005]	*0.972 [0.962, 0.992]*	1.019 [0.968, 1.073]
Daily O_3_ (in 10 µg/m^3^)	*1.034 [1.014, 1.054]*	*1.041 [1.017, 1.065]*	1.016 [0.981, 1.053]	1.065 [0.966, 1.175]
Relative humidity	1.003 [0.9996, 1.006]	1.002 [0.998, 1.006]	1.006 [0.999, 1.012]	0.997 [0.981, 1.013]
Percent of no sky view	*0.989 [0.986, 0.992]*	*0.989 [0.985, 0.992]*	*0.989 [0.983, 0.995]*	0.987 [0.972, 1.002]
NDVI (in 0.1 unit)	*0.934 [0.914, 0.955]*	*0.943 [0.918, 0.968]*	*0.911 [0.874, 0.950]*	0.954 [0.861, 1.056]
Percent of low education	0.999 [0.995, 1.004]	1.001 [0.996, 1.006]	0.996 [0.988, 1.005]	0.991 [0.970, 1.012]
Percent of non-Cantonese	0.995 [0.989, 1.002]	*0.992 [0.984, 0.999]*	1.004 [0.991, 1.016]	1.012 [0.984, 1.040]
Economically inactive	0.976 [0.870, 1.095]	0.925 [0.804, 1.064]	0.983 [0.793, 1.218]	*1.740 [1.006, 3.010]*
Unmarried	*1.352 [1.263, 1.448]*	*1.252 [1.145, 1.370]*	*1.405 [1.254, 1.575]*	*1.933 [1.480, 2.526]*
Age	*1.084 [1.080, 1.088]*	*1.064 [1.057, 1.072]*	*1.091 [1.070, 1.113]*	*1.130 [1.075, 1.188]*
Male	*0.888 [0.836, 0.943]*	*0.801 [0.743, 0.864]*	0.978 [0.876, 1.091]	*1.713 [1.255, 2.339]*

Based on each regression, the OR was used to evaluate the difference between dementia and all-cause mortality. Significant results are highlighted with italics text and with p-value < 0.05

Particularly, a 10 µg/m^3^ increase of *daily O*_*3*_ was linked to higher dementia mortality (OR: 1.034 [1.014, 1.054]), with all-cause deaths as controls. In addition, being unmarried was associated with higher dementia mortality (OR: 1.352 [1.263, 1.448]).

Furthermore, urban compactness and greenness across a sub-district were linked to lower dementia mortality. Compared with the general scenario from all-cause deaths, a 1% decrease in sky view and 0.1 increase in NDVI resulted in ORs of 0.989 [0.986, 0.992] and 0.934 [0.914, 0.955].

Finally, dementia deaths were less likely to be influenced by *daily PM*_*10*_ and *daily NO*_*x*_ compared with all-cause deaths.

### Socio-environmental impacts on old-old decedents

**Fig. 4:**
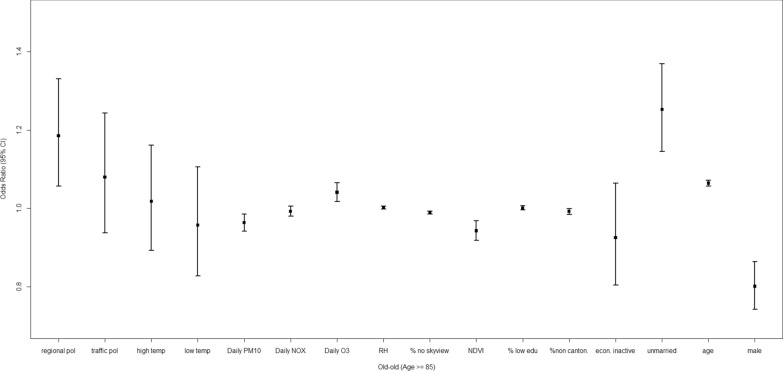
Adjusted odds ratio (OR) and the 95% confidence interval (CI) for the evaluation of relative impacts of social, built and geophysical environment on dementia mortality among old–old decedents (age > = 85)

Among the old-old decedents (Table 3), more greenness was linked to lower dementia mortality (OR: 0.943 [0.918, 0.968]). In contrast, being female was a factor associated with higher dementia mortality (Fig. 4).

Particularly, the older individuals within this group of decedents had higher dementia mortality (OR 1.064 [1.057, 1.072]. Furthermore, long-term exposure to regional air pollution and short-term exposure to tropospheric ozone were linked to higher dementia mortality, with ORs of 1.186 [1.057, 1.330] and 1.041 [1.017, 1.065]. Being unmarried was also a significant factor for dementia mortality (OR 1.252 [1.145, 1.370]). In addition, increases in *Not Cantonese %* and urban compactness across a sub-district were linked to lower dementia mortality, with ORs of 0.992 [0.984, 0.999] and 0.989 [0.985, 0.992].

Finally, dementia deaths were less likely to be linked to *daily PM*_*10*_ compared with all-cause deaths.

### Socio-environmental factors and mid-old decedents

**Fig. 5:**
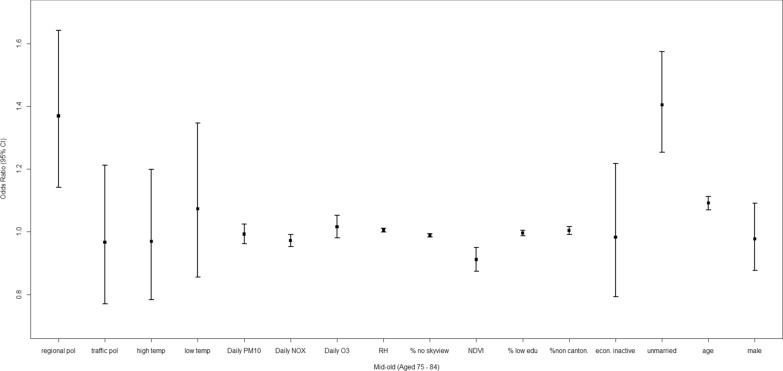
Adjusted odds ratio (OR) and the 95% confidence interval (CI) for the evaluation of relative impacts of social, built and geophysical environment on dementia mortality among mid-old decedents (age: 75–84)

The associations of socio-environmental factors with dementia mortality among the mid-old decedents were similar to those among all decedents and old-old decedents (Table 3). Specifically, long-term exposure to regional air pollution, unmarried status and older age were linked to increased dementia mortality when compared with all-cause mortality, with ORs of 1.370 [1.142, 1.643], 1.405 [1.254, 1.575], and 1.091 [1.070, 1.113]; while urban compactness and greenness were linked to lower dementia mortality, with ORs of 0.989 [0.983, 0.995] and 0.911 [0.874, 0.950]. Dementia deaths were less likely to be linked to *daily NO*_*x*_ compared with all-cause deaths (Fig. 5).

### Socio-environmental factors and young-old decedents

**Fig. 6:**
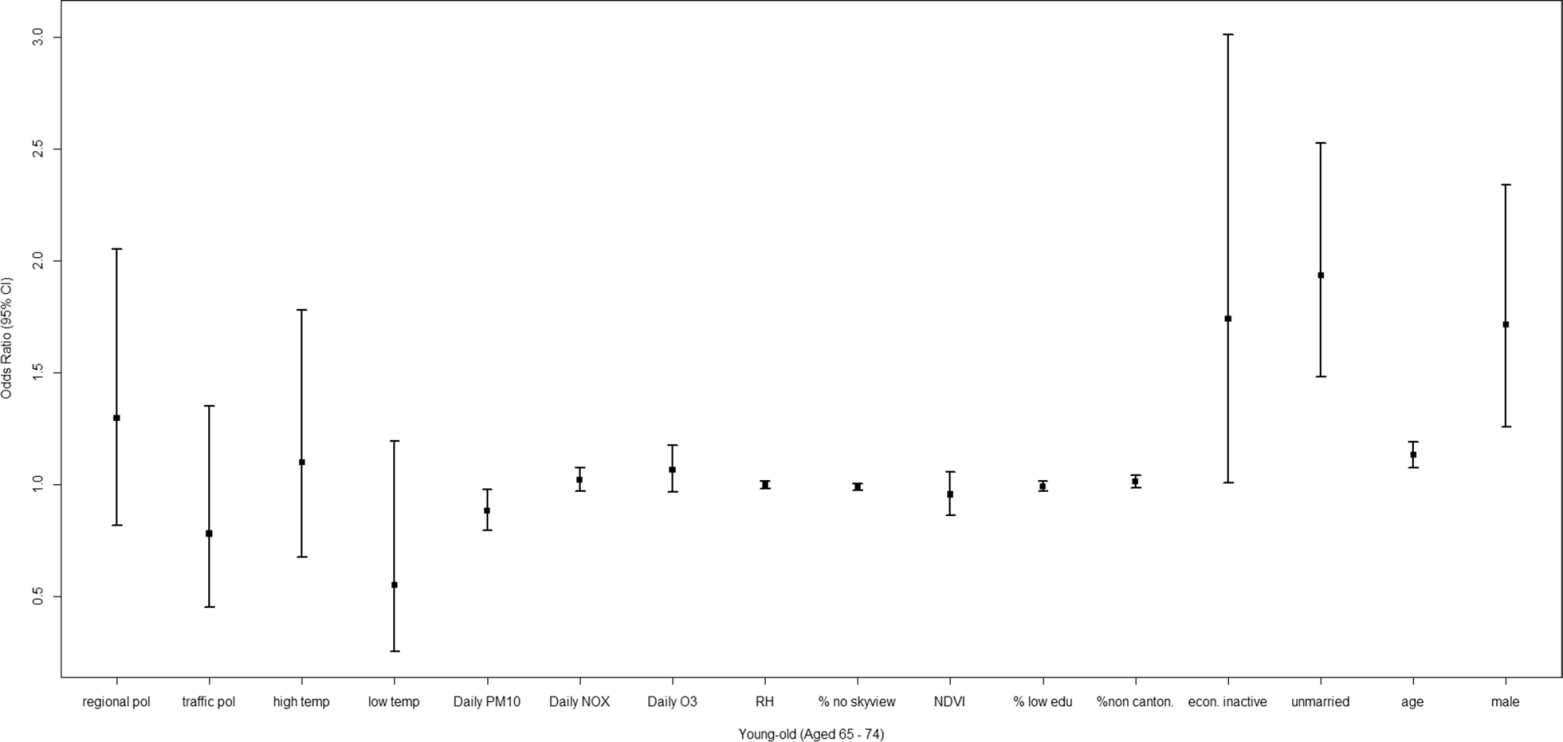
Adjusted odds ratio (OR) and the 95% confidence interval (CI) for the evaluation of relative impacts of social, built and geophysical environment on dementia mortality among young-old decedents (age: 65–74)

Among the young-old decedents (Fig. 6), only several individual-level social factors were linked to a difference in mortality risks between dementia and all-cause deaths (Table 3). Specifically, being economically inactive, unmarried, older age, and male were associated with higher dementia mortality, with ORs of 1.740 [1.006, 3.010], 1.933 [1.480, 2.526], 1.130 [1.075, 1.188], and 1.713 [1.255, 2.339]. Dementia deaths were less likely to be linked to *daily PM*_*10*_ compared with all-cause deaths.

## Discussion

### Implications of territory-wide register-based study

This study showed a significant difference in associations of social, built and geophysical environment with dementia and all-cause mortality, and this may indicate that community planning to improve social and environmental resilience among populations with dementia should be tackled with a variety of action plans.

The analyses suggested that social isolation was the biggest problem related to dementia. Specifically, being economically inactive, unmarried and older are known factors directly or indirectly related to social isolation. More people speaking a foreign language within a district could be a cultural barrier; however, this can also increase the social support within a district, enhancing community engagement and social cohesion. Furthermore, although urban compactness can be an indicator of lower environmental quality, it may somewhat reflect an environment with better social cohesion, as urban compactness in a high-rise, high-density city can refer to an urban design with high-rise building complexes that can provide better access to various facilities (e.g. community facilities and local clinics) with multifunctional land use for the local population. More specifically, a local study has found that higher accessibility of local facilities can enhance cognitive functions of older adults in Hong Kong [24]. Furthermore, a recent study in the United States has also found that areas with higher urbanization have lower dementia mortality [37]. Therefore, an alternative interpretation of the results regarding building density can be an indication of higher mortality risk from dementia in remote areas with higher potential for social isolation. Thus, a better community plan to prevent risk from dementia across a compact city (e.g. Hong Kong) should be to design the city with better social cohesion to sustain resilience among citizens. Specifically, the concept of “livability” has been widely documented, with the goal of improving the social and physical connectivity among urban residents through urban compactness, in order to maintain a lifestyle of the local population with better quality of life [22]. Based on this concept and the results of urban compactness in this study, public facilities in a high-rise, high-density environment that can maintain the social network among the local population (e.g. community centers for social services, and neighborhood facilities for family assistance) could be further improved, in order to reduce the mortality burden of older people suffering from dementia.

The present results regarding regional air pollution align with findings from previous studies that long-term air pollution can adversely influence neurodegenerative diseases [12, 13], while this study further shows adverse effects varying for those with dementia, controlling for age. Specifically, our result, although not allowing a clear causal conclusion, implies that a long-term increase in dust particles may influence oxidative stress, neuroinflammation, and neurodegeneration, as a result inducing higher dementia mortality, which is consistent with the hypotheses of a previous study [9].

The results of greenness can be interpreted in two ways. First, urban greenness has been documented to be a protective factor against dementia and related mental health diseases [46]. For example, Paul et al. [50] found that greenness could be negatively associated with dementia. Our study further implies the necessity of applying greenness as a nature-based preventive medicine against dementia mortality and related causes of death (e.g. injuries). Second, vegetation can reduce air pollution [20, 53]. This may be a modifier regarding the effect of regional air pollution on dementia mortality.

Finally, although previous studies have shown significant links between short-term environmental changes and dementia [32, 38, 66], our results indicated that the links of *daily PM*_*10*_ and *daily NO*_*x*_ with dementia are not as strong as those for all-cause mortality, at least among the local population in Hong Kong. This could be due to a short-term increase in dust particles (e.g. PM_10_) inhaled into the lungs inducing a stronger risk of having cardiorespiratory illness than dementia. However, the stronger association between *daily O*_*3*_ and dementia mortality implies that toxic gas (e.g. ozone) may influence oxidative stress in the short-term, resulting in higher dementia mortality than that of the other diseases. Furthermore, the above results also imply that vulnerable population segments such as dementia patients might have less resilience and preparedness to protect themselves from any short-term and critical changes caused by extremes in environmental quality [65]. In fact, the above results could be partially explained by dementia patients’ need for institutional care [22], and their lower awareness of environmental changes [32]. This could also be linked to the assumption of social cohesion above, such that if health education to improve knowledge, attitudes, and practices could be provided to caregivers [55], it might help dementia patients better cope with the above environmental hazards from short-term changes caused by extremes in environmental quality. However, if caregivers could not deliver immediate support to the vulnerable population, environmental risk might cause fatal influences on dementia patients. It is also important to note that environmental changes associated with temperature and air quality can induce severe health risk in normal people within a matter of hours [39, 74]. For the case of dementia mortality, this effect could be magnified.

### Limitations

Data on the spatial mobility of each decedent were unavailable in this study. Several studies have suggested that socio-environmental exposure can be a spatiotemporal component among the urban population, while variations in socio-environmental exposure from multiple locations within cities can affect the degree of impact on each citizen. While the census registry, due to its nature, is not useful to represent this spatiotemporal phenomenon, such spatiotemporal bias is not as significant as in studies representing the working population and adolescents, since persons suffering with dementia are usually older people with lower mobility. In addition, older people usually prefer to stay in their own neighborhoods for daily living, and this suggests that our approach may still be appropriate. For future study, a GPS tracker could be used to enhance the measurement of each subject, but this approach can only target the surviving population, not the decedents.

Another limitation is that the database lacks information on specific types of dementia for many cases, and longitudinal information related to co-morbidities or survival period was not provided by the database either. Two diseases—Alzheimer’s disease and first stroke—may have some overlap in the cause of death resulting from vascular dementia, and vascular dementia can be a result of ischemic stroke [36]. Furthermore, dementia mortality could be a progressive consequence from injury and genetic issues [26, 42] but cross-sectional data without information related to co-morbidities could not address the above question. Therefore, more comprehensive pathways regarding burden of diseases will be provided if longitudinal information on co-morbidities can be included in the analyses.

Furthermore, this study used community-level information (e.g. average NDVI) to represent residential location for the estimation of the possible influence of urban characteristics on dementia mortality, due to the limitation of register-based mortality data. However, some studies have suggested that since older adults are less mobile, their movements should be analyzed with a shorter spatial distance [10], such as 500 m from their homes. Therefore, future studies with population-based cohort data and finer spatial information could be developed to estimate the association at various scales (e.g. 500 m radius versus TPU-level) between relevant urban factors and dementia mortality through hierarchical modelling or multilevel regression (e.g. a linear mixed effect model). However, the above approach is limited by data collection, especially because the sample size is usually not as large as register-based data from government. Therefore, the approach used in the present study is still appropriate.

Additionally, using community-level data may cause a Modifiable Unit Areal Problem (MAUP) due to grouping data with different zones [27]. For this study, we used TPU, which is one of the finest spatial units for planning in Hong Kong, for community health analyses. Despite the possibility of MAUP, the information from this study could still be useful for health resource allocation in a local context, because local town planners and health officials have commonly used TPU for neighborhood health planning. To minimize MAUP in the future, further studies could consider disaggregating and re-aggregating community-level data for sensitivity analyses.

In this study, we developed our model based on previous local studies [11, 23, 28, 68] which reported that the strongest influences on mortality occur at lag 0 days. Therefore, we only included a lag 0 day scenario for temporally varying measures. However, studies from other locations showed that environmental exposures such as temperature might influence local health over a longer period [44]. Therefore, more lag days with a moving average should be considered in future studies.

Finally, this study followed other local studies [24]; Ho et al. [29] in assuming that the older adults may have been staying in their locations of residence for a long period. However, some of the residents may have been moved to another location such as long-term health care facilities. As this is a limitation of register-based data, future studies should apply a cohort design with more information related to residential address for a comparison.

## Conclusions

This territory-wide register-based study indicated a difference in the association of various factors with dementia mortality among different ages in Hong Kong. Specifically, social isolation and regional air pollution may have stronger links to dementia mortality. A short-term increase in *daily O*_*3*_ is also associated with higher dementia mortality within a day. A comprehensive framework for community health planning should be conducted based on the relative impacts of various diseases in order to reduce vulnerability at all ages.

## Data Availability

All data generated or analyzed during this study are available from the corresponding author on reasonable request.
